# High-risk HPV prevalence in the Czech cervical cancer screening population: a comparison of clinician-collected and self-collected sampling

**DOI:** 10.1093/eurpub/ckaf045

**Published:** 2025-04-07

**Authors:** Hana Jaworek, Ondrej Bouska, Pavla Kourilova, Marian Hajduch, Vladimira Koudelakova

**Affiliations:** Institute of Molecular and Translational Medicine, Faculty of Medicine and Dentistry, Palacky University Olomouc, Olomouc, The Czech Republic; Institute of Molecular and Translational Medicine, University Hospital, Olomouc, The Czech Republic; Institute of Molecular and Translational Medicine, Faculty of Medicine and Dentistry, Palacky University Olomouc, Olomouc, The Czech Republic; Institute of Molecular and Translational Medicine, Faculty of Medicine and Dentistry, Palacky University Olomouc, Olomouc, The Czech Republic; Institute of Molecular and Translational Medicine, Faculty of Medicine and Dentistry, Palacky University Olomouc, Olomouc, The Czech Republic; Institute of Molecular and Translational Medicine, University Hospital, Olomouc, The Czech Republic; Cancer Research Czech Republic, Olomouc, The Czech Republic; Institute of Molecular and Translational Medicine, Faculty of Medicine and Dentistry, Palacky University Olomouc, Olomouc, The Czech Republic; Institute of Molecular and Translational Medicine, University Hospital, Olomouc, The Czech Republic

## Abstract

The prevalence of high-risk human papillomavirus (hrHPV) types varies across countries, making it essential to estimate prevalence using nationwide samples. Data on hrHPV prevalence in the Czech Republic are very limited. This study aimed to determine the prevalence of various hrHPV types in an unselected screening population of Czech women aged 30–65 years, using paired clinician-obtained cervical swab (CS) and self-collected cervicovaginal swabs (CVS). A total of 1026 eligible women were recruited into two study arms. In arm A, the *digene*^®^ HC2 DNA Collection Device was used for both CS and CVS. In arm B, the Evalyn Brush was used for CVS, while the Cervex Brush was used for CS. All samples were tested for hrHPV using the *digene*^®^ HC2 High-Risk HPV DNA Test and genotyped with the PapilloCheck^®^ HPV-Screening assay. The overall hrHPV prevalence was 14.8%, based on positive results from either CVS or CS samples. hrHPV positivity was detected in 10.8% of clinician-obtained CSs and 11.8% of self-collected CVSs. A combined analysis of CS and CVS samples identified the five most prevalent hrHPV genotypes: HPV16, HPV31, HPV39, HPV56, and HPV68. The comparison of hrHPV detection in paired CS and CVS samples showed an overall concordance of 93%. These findings highlight the importance of detecting hrHPV genotypes alongside conventional Pap testing in national cervical screening programs. Furthermore, the results confirm that self-sampling kits represent a suitable alternative to clinician-collected samples.

**Clinical trials registration**  ClinicalTrials.gov Identifier (NCT04133610)

## Introduction

Cervical cancer, despite being a preventable disease, remains the fourth most common cancer and the fourth leading cause of death among women worldwide [1]. Despite high overall coverage of cervical cancer screening, Central and Eastern Europe report the highest incidences of cervical cancer in Europe, often with diagnoses at advanced stages, particularly among older women, who also show a significant decline in screening coverage. The incidence of cervical cancer in Czech Republic peaks between the ages of 40 and 45. This high incidence may also be partly attributed to the use of cytology, a screening method with lower sensitivity [1–4]. Therefore, transitioning from opportunistic to organized, population-based Human Papillomavirus screening programs is essential. Other European regions with successful prevention programs are already integrating population-based HPV screening and self-sampling methods into screening strategies [5].

When compared to conventional cytology, HPV testing is more sensitive and reproducible, which enables extended screening intervals and the analysis of self-collected vaginal samples. Self-sampling is a safe and straightforward method that allows women who do not participate in clinician-based screening to comfortably access the screening test [5, 6]. In addition to providing physical and emotional comfort, self-collected samples have shown comparable diagnostic accuracy for cervical cancer and high-grade cervical intraepithelial neoplasia (≥CIN 2) as clinician-sampled CSs when HPV detection is employed [7].

The Czech Republic has a well-established national vaccination (for girls since 2012, gender-neutral since 2018) and cervical cancer screening program (since 2008) [8]. The screening program is based on annual cytological examinations and is fully covered by health insurance for all women aged 15 and older. Over time, the screening program has undergone three major updates. Since 2014, the first introduced personalized invitations for non-attenders aged 25 and older to boost participation. In 2021, the strategy was further refined to enhance early detection, particularly in the most affected age groups, by incorporating one-time HPV co-testing at ages 35 and 45 alongside annual cytology screening. In 2024, the program was further expanded to include HPV co-testing for women at age 55, strengthening efforts to detect cervical cancer in later life stages [4, 9]. Currently, only limited data concerning HPV prevalence in Czech women over 30 years of age are available.

This study aimed to evaluate HPV prevalence in the Czech cervical cancer screening population and compare the cervical clinician-collected high-risk HPV (hrHPV) tests with those from a cervicovaginal self-collected HPV test. Additionally, we aimed to compare the effectiveness of different sampling devices, including the *digene*^®^ Collection Device (QIAGEN GmbH, Hilden, Germany) for both clinician- and self-sampling, the Cervex Brush (Rovers Medical Devices B.V., Oss, the Netherlands) for CSs, and the Evalyn Brush (Rovers Medical Devices B.V.) for self-sampled cervicovaginal swabs (CVS).

## Methods

### Study design

The study was carried out in cooperation with three gynecological centers across the Czech Republic, with one in the Olomouc region and two in the Moravian-Silesian region, from November 2018 to August 2019. The recruited participants represented sexually active women between 30 and 65 years of age who were undergoing routine primary screening with a Pap test at their gynecologists. Exclusion criteria for the study included no sexual intercourse experience, pregnancy, HPV vaccination, increased risk of bleeding, CIN or cervical carcinoma in anamnesis, cervical conization, or hysterectomy.

Sample size was estimated using G*Power software. When we calculated with 80% power and a 5% probability of Type I error, ref. proportion 12.6% and the effect size −3%, the total required sample size was 877. Since we expected the proportion of patients with normal cytological finding to be 95%, the total number of patients required for the study was 924. With an estimated 10% dropout rate, at least 1027 women needed to be approached.

A total of 1047 women were enrolled in the study; seven women did not sign the informed consent, and 14 women were younger than 30 years. Overall, 1026 women who met the entry criteria were included in the research. A total of three samples were collected from each study participant during one gynecological visit. A cervicovaginal self-sample and a clinician-collected CS were obtained for HPV testing, while another clinician-obtained CS was used for cytology examination. The cervical cytology screening test was performed at EUC Laboratory CGB, Inc. (Ostrava, Czech Republic).

**Figure 1.**
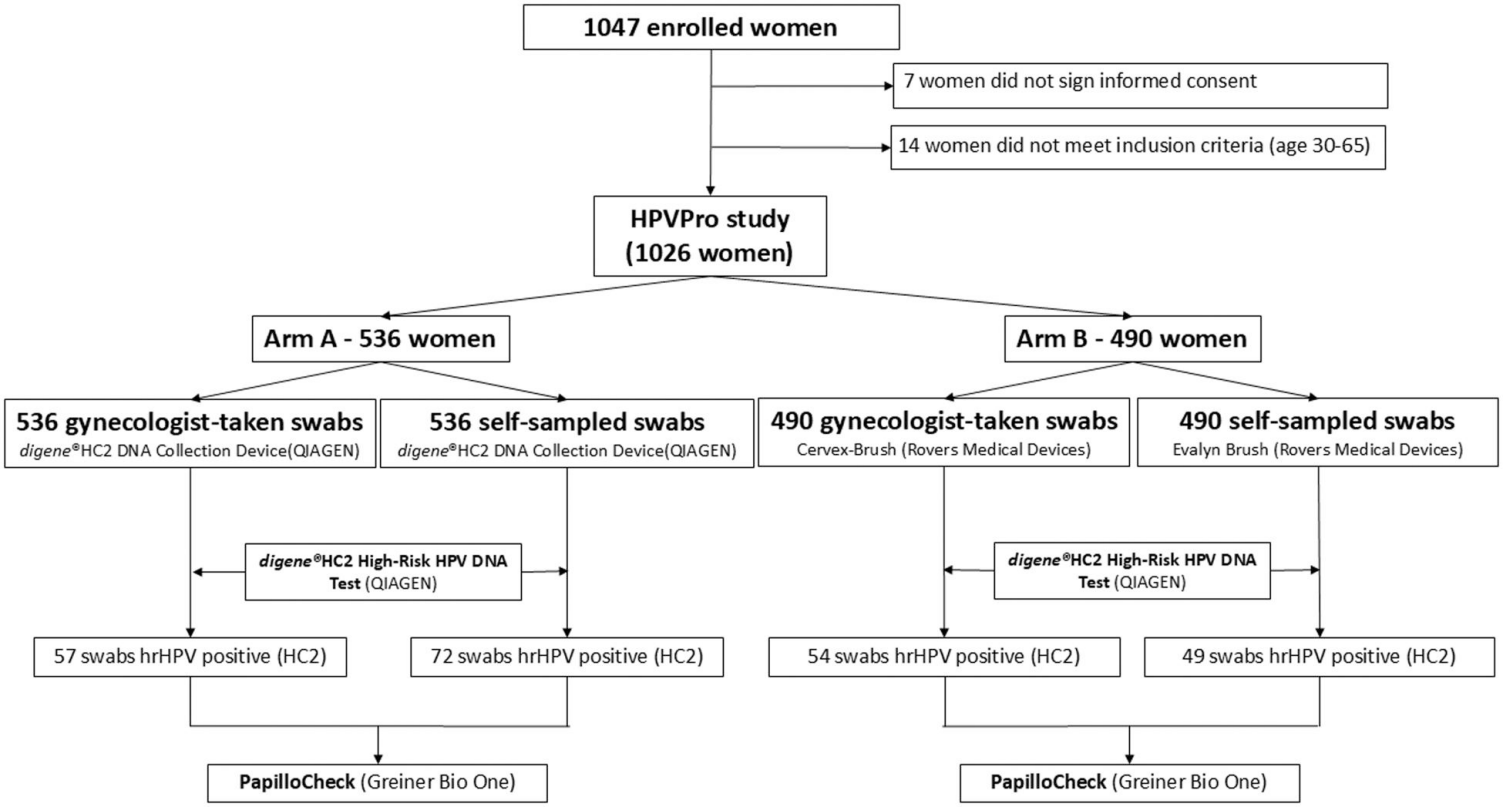
Flow diagram of women in the study arms.

The study was conducted in two arms (Fig. 1). Arm A involved self-sampling and physician sampling using the *digene*^®^ HC2 DNA Collection Device (QIAGEN GmbH). Arm B involved self-sampling using the Evalyn Brush device (Rovers Medical Devices B.V.) and clinician sampling using the Cervex Brush (Rovers Medical Devices B.V.). Cervical swabs (CSs) and CVSs were collected in parallel in both study arms.

All of the study participants provided written informed consent. This study was performed in compliance with the Helsinki Declaration according to the study ethics proposal approved by the Ethics Committee of the Faculty of Medicine and Dentistry at Palacky University and the University Hospital in Olomouc (protocol no. 97/13).

### Clinical specimen collection and processing

Women first self-collected a CVS, after which two CSs (for cervical cytology screening and HPV testing) were taken by a gynecologist. Following sampling, the *digene*^®^ HC2 DNA Collection Device was rinsed in specimen transport media (STM), which was provided as part of the sampling kit. Prior to use, the Cervex Brush was rinsed in ThinPrep^®^ Pap Test PreservCyt^®^ Solution (Hologic, Inc., Marlborough, MA, USA), while the Evalyn Brush device was sealed in a dry state inside its original packaging with a cap and sent to the laboratory. All samples were stored and transported at room temperature and then processed in line with the directions for *digene*^®^ HC2 high-risk HPV DNA Test (QIAGEN GmbH), herein referred to as “HC2” testing.

At the laboratory, 800 µl of STM media was required for HC2 testing, while 100 µl of STM media was used for DNA extraction. Evalyn Brush heads were suspended in 3 ml of phosphate-buffered saline (PBS) solution, 1 ml of PBS was required for HC2 testing and 300 µl of PBS media was used for DNA extraction. For the swabs collected using the Cervex Brush, 4 ml of ThinPrep^®^ Pap Test PreservCyt^®^ Solution was required for HC2 testing after the sample conversion step, which was performed using the *digene*^®^ HC2 Sample Conversion Kit (QIAGEN GmbH), and 1 ml of solution was used for DNA extraction.

### HPV DNA detection

All of the samples were tested for the presence of hrHPV DNA using the HC2 test following the manufacturer's recommended protocol. For all of the HC2 samples which returned a positive result, DNA was extracted using the QIAamp DNA Micro Kit (QIAGEN GmbH) and genotyped using the PapilloCheck^®^ HPV-Screening assay (Greiner Bio-One, Frickenhausen, Germany), as described in a previous article [10].

### Statistical analysis

Statistical analyses were performed using R, ver. 3.5.2 (Core Team, 2018), with the threshold for significance set as *P* < .05. Comparisons between groups were performed using the Wilcoxon test and Pearson's chi-square test. Concordance was computed using a binomial test. The same test has been used for testing a difference from constant proportion. To reveal the difference between two methods of sample collection from the same subject McNemar test of symmetry was applied.

## Results

Paired CSs and CVSs for HPV testing were collected from 1026 women during regular gynecological check-ups. Only women in the recommended cervical cancer screening age range (30–65 years) were included in this study. The median age of the women participating in our study was 44.33 years. Most women, i.e. 95% (975/1026), had normal cytological findings (NILM), while atypical cytology findings (≥ASC-US, atypical squamous cells of undetermined significance) were found in 5% (51/1026) of the participants (Table 1).

**Table 1.** digene^®^HC2 high-risk HPV DNA test results for paired samples by age, cytology, and sampling type

	CS, *N* (%)			CVS, *N* (%)			CS/CVS concordance	
	HC2 positive	HC2 negative	Total	HC2 positive	HC2 negative	Total	*N*	Concordance (95% Cl)
**Total**	111 (10.8%)	915 (89.2%)	1,026 (100%)	121 (11.8%)	905 (88.2%)	1026 (100%)	954/1026	0.930 (0.912–0.945)
**Age (years)**								
(30; 40)	53 (47.7%)	291 (31.8%)	344 (33.5%)	55 (45.5%)	289 (31.9%)	344 (33.5%)	314/344	0.913 (0.878–0.940)
(40; 50)	42 (37.8%)	390 (42.6%)	432 (42.1%)	46 (38%)	386 (42.7%)	432 (42.1%)	404/432	0.935 (0.908–0.957)
(50; 60)	14 (12.6%)	194 (21.2%)	208 (20.3%)	18 (14.9%)	190 (21%)	208 (20.3%)	196/208	0.942 (0.901–0.970)
(60; 65)	2 (1.8%)	40 (4.4%)	42 (4.1%)	2 (1.7%)	40 (4.4%)	42 (4.1%)	40/42	0.952 (0.838–0.994)
Average age (years)	41.17	44.59	44.22	41.85	44.53	44.22	–	–
Median age (years)	40.19	43.71	43.33	40.60	43.60	43.33	–	–
**Cytology**								
NILM	89 (80.2%)	886 (96.8%)	975 (95%)	100 (82.6%)	875 (96.7%)	975 (95%)	906/975	0.929 (0.911–0.945)
ASC-US/AGC-NOS	12 (10.8%)	27 (3%)	39 (3.8%)	12 (9.9%)	27 (3.0%)	39 (3.8%)	37/39	0.949 (0.827–0.994)
LSIL	8 (7.2%)	2 (0.2%)	10 (1.0%)	7 (5.8%)	3 (0.3%)	10 (1.0%)	9/10	0.900 (0.555–0.997)
ASC-H	2 (1.8%)	0 (0%)	2 (0.2%)	2 (1.7%)	0 (0%)	2 (0.2%)	2/2	1.000 (0.158–1.000)
≥ASC-US	22 (19.8%)	29 (3.2%)	51 (5%)	21 (17.4%)	30 (3.3%)	51 (5.0%)	48/51	0.941 (0.838–0.988)

ASC-US, atypical squamous cells of undetermined significance; ASC-H, atypical Squamous Cells, HSIL cannot be excluded; AGC-NOS, atypical glandular cells, not otherwise specified, CS, cervical swab; CVS, cervicovaginal swab; LSIL, low-grade squamous intraepithelial lesions; NILM, negative for intraepithelial lesions or malignity.

Overall, hrHPV positivity detected by the HC2 test, combining both CSs and CVSs, was observed in 14.8% (152/1026) of cases. The comparison of hrHPV detection in paired samples showed an overall concordance of 93% (954/1026) (Table 2). Clinician-collected CSs were positive in 10.8% (111/1026) of cases, while self-collected CVSs showed hrHPV positivity in 11.8% (121/1026) of cases (Table 1).

**Table 2.** Comparison of hrHPV positivity (*digene^®^* HC2 High-Risk HPV DNA Test) in self-sampled CVSs and physician-obtained CSs in both study arms

CSs	CVSs	Both arms (A + B)	**Arm A** ^a^	**Arm B** ^b^
**Total**		1,026 (100%)	536 (100%)	490 (100%)
HC2 positive	HC2 positive	80 (7.8%)	47 (8.8%)	33 (6.7%)
HC2 positive	HC2 negative	31 (3.0%)	10 (1.9%)	21 (4.3%)
HC2 negative	HC2 positive	41 (4.0%)	25 (4.7%)	16 (3.3%)
HC2 negative	HC2 negative	874 (85.2%)	455 (84.7%)	419 (85.7%)
**Overall HC2 positive (CS and/or CVS)**		152 (14.8 %)	82 (15.3%)	70 (14.3%)
**Concordance of CS and CVS**		954 (93.0 %)	502 (93.7%)	452 (92.2%)
**HC2 positive without cross-reaction** ^c^		92 (9.0 %)	52 (9.7%)	40 (8.2%)

CS, cervical swab; CVS, cervicovaginal swab; hrHPV, high-risk human papillomavirus; HC2, *digene^®^* HC2 high-risk HPV DNA test.

a *digene* ^®^ HC2 DNA Collection Device was used for both CS and CVS.

b The cervex-brush and Evalyn Brush were used for CS and CVS sampling, respectively.

c Based on the results of the PapilloCheck^®^ HPV-screening assay.

Age dependent analysis of hrHPV prevalence revealed similar trends in both sample types. In CSs, prevalence ranged from 47.7% in women aged 30–40 years to 1.8% in those aged 60–65 years. Similarly, in CVSs, hrHPV prevalence ranged from 45.5% in women aged 30–40 years to 1.7% in those aged 60–65 years (Table 1). Additionally, hrHPV-positive women were significantly younger than hrHPV-negative women (CS median age: 40.2 vs. 43.7 years, *P* < .001; CVS median age: 40.6 vs. 43.6 years, *P* < .001).

HC2 detection of hrHPV in women with NILM was positive in 9.1% (89/975) of CSs and 10.3% (100/975) of CVSs. Among women with ASC-US/AGC-NOS findings, hrHPV detection was positive in 30.8% (12/39) of CSs and 30.8% (12/39) of CVSs. In women diagnosed with LSIL (low-grade squamous intraepithelial lesions), 80% (8/10) of CSs and 70% (7/10) of CVSs tested hrHPV positive. Notably, all women with ASC-H (Atypical Squamous Cells, HSIL cannot be excluded) had hrHPV-positive results in both sample types (2/2) **(**Table 1**)**.

Among the 72 women with discordant results in paired samples, 31 had hrHPV-positive CSs only, while 41 had hrHPV-positive CVSs only (Table 2). Most of the discordant results, 95.8% (69/72), were observed in women with NILM cytological findings. In contrast, only 4.2% (3/72) of discordant results were found among women diagnosed with ≥ASC-US, and no discordant results were observed in women with ASC-H.

HrHPV prevalence in the clinician-taken CSs was consistent across both study arms (10.6% vs 11.0%, *P* = .913). However, there was a slight difference in hrHPV prevalence among the self-sampled CVSs between the study arms. More specifically, the first arm demonstrated a slightly higher percentage of hrHPV positivity in the CVSs than the second arm (13.4% vs 10.0%, *P* = .111). The results of hrHPV positivity of paired CSs and CVSs were statistically significantly different in study arm A, with 6.5% (35/536) discordant pairs and more positives only in CVS samples [odds ratio = 2.5, McNemar test, p(A) = 0.018]. However, the difference between CVS and CS in both study arms combined (A + B) and in arm B alone (see Table 2) was not statistically significant [McNemar test, p(A + B) = 0.289, p(B) = 0.511]. Nevertheless, the proportion of discordant pairs and the corresponding odds ratio in arms A + B and B were not high enough to reject the hypothesis of no difference between CS and CVS samples with a reasonably high power of the test.

To determine whether this observed between-arm difference in hrHPV positivity in CVSs could be explained by the different devices employed in the two study arms, we compared HC2 signal strength values, i.e. relative light units/cut-off (RLU/CO), which can indicate viral load [11]. The results of the analysis revealed that there was no significant difference in the median RLU/CO values (11.0 vs 9.38, *P* = .301) for the CVS samples collected by the *digene*^®^ HC2 DNA Collection Device (arm A) and the Evalyn Brush (am B). Furthermore, no significant differences were observed between CVS samples taken using the *digene*^®^ HC2 DNA Collection Device (arm A) and the Cervex Brush (arm B) (median RLU/CO values 21.2 vs 59.6, *P* = .301). The only significant difference was observed between CSs and CVSs, with CSs showing higher median RLU/CO values (combined for arms A and B) than CVSs (median RLU/CO 34.7 vs 9.53, *P* = .002).

Genotyping of the HC2 hrHPV-positive samples revealed that 49.6% (55/111) of hrHPV-positive CSs and 42.1% (51/121) of hrHPV-positive CVSs samples had a single hrHPV infection. Multiple hrHPV type infections were observed in 33.3% (37/111) of CSs and 36.4% (44/121) of CVSs (Table 3). HPV16, HPV18, and other hrHPV genotypes were detected in 16.2% (18/111), 5.4% (6/111), and 61.3% (68/111) of CSs, respectively. An analysis of the hrHPV types present in CVSs revealed that HPV16, HPV18, and other hrHPVs were detected in 10.7% (13/121), 4.1% (5/121), and 63.6% (77/121) of the samples, respectively. Type-specific HPV prevalence is summarized in Table 3. In 17.1% (19/111) of the CS samples, either no HPV type was detected (*n* = 9) or a positive result was likely caused by cross-reactivity of HC2 with non-targeted HPV genotypes (*n* = 10). The CVS samples showed similar results, more specifically, nine samples were negative for any HPV type while 13 most likely returned a positive result due to cross-reactivity. The most frequently detected HC2 non-targeted genotype was HPV53 in CSs and HPV42 in CVSs (Table S1).

**Table 3.** The distribution of HPV genotypes tested using the PapilloCheck^®^ HPV-screening assay.

	CSs, *N* (%)	CVSs, *N* (%)
**HC2 hrHPV positive samples (total)**	111	121
Single hrHPV infection	55 (49.6%)	51 (42.1%)
Multiple infections^a^	37 (33.3%)	44 (36.4%)
N/cross-reaction^b^	19 (17.1%)	22 (18.2%)
PapilloCheck failure	–	4 (3.3%)
HPV16	18 (16.2%)	13 (10.7%)
HPV18	6 (5.4%)	5 (4.1%)
HPV31	17 (15.3%)	15 (12.4%)
HPV33	6 (5.4%)	4 (3.3%)
HPV35	3 (2.7%)	2 (1.7%)
HPV39	9 (8.1%)	12 (9.9%)
HPV45	6 (5.4%)	6 (5%)
HPV51	6 (5.4%)	11 (9.1%)
HPV52	10 (9%)	5 (4.1%)
HPV56	13 (11.7%)	18 (14.9%)
HPV58	1 (0.9%)	4 (3.3%)
HPV59	4 (3.6%)	6 (5%)
HPV68	16 (14.4%)	18 (14.9%)

a Multiple infections—includes samples with multiple hrHPV genotypes detected or samples with a single hrHPV genotype detected along with additional HPV genotypes (non-targeted by *digene*^®^ HC2 High-Risk HPV DNA Test) by PapilloCheck^®^ HPV-Screening assay.

b *N*/cross-reaction—includes samples where no HPV genotype was detected or only *digene*^®^ HC2 High-Risk HPV DNA test non-targeted HPV genotype was detected using PapilloCheck^®^ HPV-Screening assay.

## Discussion

The burden of cervical cancer is increasing on a global level, and can be perceived even in countries with well-established, cytology-based screening programs. Therefore, the adoption of hrHPV testing is crucial to ensuring effective preventative measures [5]. In addition to being more effective in identifying cases of precancer and cancer than cytology, [6, 12] hrHPV testing can be performed on self-collected samples. In contrast, cytology on self-samples has previously shown poor accuracy [7, 13].

The main objective of this study was to determine the recent prevalence of hrHPV in the Czech cervical cancer screening population. This study is the first to focus on a screening population of unselected women between 30 and 65 years of age in the Czech Republic. Paired cervical and CVSs were analyzed from all of the study participants. We found a prevalence rate for hrHPV of 14.8% in the study population based on a positive result in either the cervicovaginal or CSs. HrHPV positivity was observed in 10.8% of clinician-obtained CSs and 11.8% of self-collected CVSs. The prevalence of hrHPV among women with NILM was lower in both CSs (9.1%) and CVSs (10.3%) compared to the average HPV prevalence reported for Central and Eastern Europe [12.6%, exact binomial test, p(CSs) < 0.001, p(CVSs) = 0.026] by Poljak *et al.* in 2013 [14]. It was also lower than the 14.2% prevalence reported for Europe overall in a meta-analysis by Bruni *et al.*, published in 2010 [15]. The observed prevalence was even lower than that reported in the only other Czech population study, published by Tachezy *et al.* in 2013, which found an hrHPV prevalence of 15.6% among women with NILM [16].

The highest hrHPV prevalence was observed among women between 30 and 40 years of age. As expected, a correlation was found between hrHPV prevalence and age. Similar to what has been reported in other studies, hrHPV prevalence decreased with increasing age in the study population analyzed in the presented research [8, 17, 18] The majority of women had normal cytological findings, with only 5% showing abnormal cytology findings (≥ASC-US); this percentage is in line with the worldwide data [19]. As expected, hrHPV prevalence increased with the severity of cytological findings among the study population, from 9.1% in women with normal cervical cytology to 100% in women with ASC-H [8]. Moreover, the most prevalent hrHPV type detected in CSs was HPV16, which is in accordance with previous evidence. Other frequently detected hrHPV types were HPV31, HPV68, HPV56, HPV52, and HPV39, which differ from the most frequently found genotypes worldwide [8, 17, 20] and from data from the Central and Eastern Europe, [14] as well as from the only other study in the Czech Republic [16]. This discrepancy could be explained by the fact that the prevalence of hrHPV types varies by region and country, [8, 17] and may depend on the hrHPV detection method and collection device used [7, 21]. For instance, signal-based HPV tests performed on self-collected samples are less sensitive and specific than those performed on clinician-obtained CSs. Conversely, PCR-based (polymerase chain reaction) tests show similar sensitivity for both sample types [22]. The lower sensitivity of signal-based assays on self-collected samples is due to lower hrHPV DNA loads in the vagina, which may fall below the detection threshold of these assays but are still detectable by PCR tests. The lower sensitivity of signal-based methodologies may also be due to noticeable levels of cross-reaction with low-risk HPV types and high-risk HPV particles in the vagina that have not caused precancers, such as CIN2, or resulted in tumorigenesis [23].

The presented research employed two different devices for the collection of cervical as well as CVSs. The *digene*^®^ HC2 DNA Collection Device and the Cervex Brush were used to collect CSs. Although no statistically significant differences were observed between these two approaches for obtaining CSs, the CSs collected using the Cervex Brush demonstrated median RLU/CO values that were more than two-fold greater than what was measured for CS samples from the digene^®^ HC2 DNA Collection Device (59.6 vs. 21.2). The effectiveness of the Cervex Brush in collecting endocervical cells, which are more likely to be infected by HPV, has been described in the literature [24, 25]. No difference was observed between the *digene*^®^ HC2 DNA Collection Device and the Evalyn Brush, as the samples associated with these two devices showed comparable median RLU/CO values. A significant difference in the values associated with all the collected CSs and CVSs was noticed, with the CSs exceeding the median RLU/CO value measured for CVSs by almost four times. This finding aligns with the supposition that self-collected materials for hrHPV tests could show lower specificity and sensitivity than clinician-collected CSs due to the inadequate collection of cells from the lower vagina [23]. Hence, self-collected swabs should be subjected to HPV detection assays based on PCR, which can amplify the collected genetic material, to ensure reliable performance [7]. Self-sampling coupled with hrHPV PCR detection is used in cervical screening across several countries, [3] and seems promising for engaging Czech women who have not yet attended a cervical screening program [4].

In conclusion, this is the first study to evaluate hrHPV prevalence among unselected Czech women within the cervical cancer screening population, specifically those aged 30–65 years. The overall prevalence of hrHPV infection was 10.8% in clinician-obtained CSs and 11.8% in self-collected CVSs. Our findings demonstrate a high concordance between clinician-collected and self-collected samples, supporting the reliability of self-sampling for HPV testing. These results highlight the potential of self-sampling and primary HPV testing in improving cervical cancer screening in the Czech Republic. Evidence from other countries suggests that adopting this approach could enhance screening effectiveness and help reduce cervical cancer incidence.

## Supplementary Material

ckaf045_Supplementary_Data

## Data Availability

The data that support the findings of this study are not openly available due to reasons of sensitivity and are available from the corresponding author upon reasonable request. Data are located in controlled access data storage at Palacky University Olomouc. Key points The prevalence of hrHPV infection in Czech women is 10.8% in clinician-obtained cervical swabs and 11.8% in self-collected cervicovaginal swabs. Concordance of clinician-obtained cervical swabs and self-collected cervicovaginal swabs was 93%. Clinician-obtained cervical swabs showed significantly higher viral load compared to self-collected swabs. Both cervical swab devices, as well as both self-sampling devices, were comparable.
