# Supplementary material for: High-risk HPV prevalence in the Czech cervical cancer screening population: a comparison of clinician-collected and self-collected sampling
Source: Eur J Public Health. 2025 Apr 7;35(4):760–5. doi: 10.1093/eurpub/ckaf045 (PMC12311329; doi:10.1093/eurpub/ckaf045)
Supplement: ckaf045_Supplementary_Data [file ckaf045_supplementary_data.docx]

**Table S1** Non-target HC2 HPV genotypes detected in *digene*®HC2 High-Risk HPV DNA Test positive samples using the PapilloCheck® HPV-Screening assay

| **HPV type** | **CSs** (N=10*) | **CVSs** (N=13^$^) | **Total** (N=23) |
| --- | --- | --- | --- |
| HPV6 | 1 | 0 | 1 (4.3%) |
| HPV40 | 0 | 1 | 1 (4.3%) |
| HPV42 | 2 | 6 | 8 (34.8%) |
| HPV43 | 0 | 1 | 1 (4.3%) |
| HPV44/55 | 1 | 0 | 1 (4.3%) |
| HPV53 | 4 | 3 | 7 (30.4%) |
| HPV66 | 2 | 0 | 1 (4.3%) |
| HPV70 | 0 | 4 | 4 (17.4%) |
| HPV73 | 2 | 0 | 2 (8.7%) |
| HPV82 | 2 | 1 | 3 (13.0%) |

CSs — cervical swabs, CVSs — cervicovaginal swabs; *two non-target genotypes were found in 4 CS samples; ^$^two non-target genotypes were found in 3 CVS samples
